# Redeployment of psychiatrist trainees during the COVID-19 pandemic: evaluation of attitude and preparedness

**DOI:** 10.5339/qmj.2021.64

**Published:** 2021-10-28

**Authors:** Ovais Wadoo, Sami Ouanes, Areej Al Siaghy, Mohamed H.M.O. Hassan, Yuri Zoghbi, Majid Alabdulla

**Affiliations:** ^1^Department of Psychiatry, Hamad Medical Corporation, Doha, Qatar E-mail: souanes@hamad.qa

**Keywords:** psychiatry, trainees, residents, fellows, COVID-19, pandemic, redeployment

## Abstract

**Background:** The coronavirus disease-2019 (COVID-19) pandemic has imposed an unprecedented strain on healthcare systems worldwide. In response, psychiatrist trainees were redeployed from their training sites to help manage patients with COVID-19. This study aimed to examine the attitude of psychiatrist trainees toward redeployment to COVID-19 sites and their perceived preparedness for managing physical health conditions during redeployment. **Methods:** A cross-sectional researcher-developed online survey was administered among psychiatrist trainees in May 2020 at the Department of Psychiatry, Hamad Medical Corporation, Qatar. **Results:** Of the 45 psychiatrist trainees, 40 (88.9%) responded to the survey. Most trainees reported being comfortable dealing with chronic medical conditions, but less so with acute life-threatening medical conditions. Half reported feeling anxious about redeployment, and most felt the need for additional training. We found that trainees’ perceived redeployment preparedness was significantly associated with their level of postgraduate training and the time since and duration of their last medical or surgical training. **Conclusion:** Adequate preparation and training of psychiatrist trainees is important before redeployment to COVID-19 sites to ensure that they can effectively and safely manage patients with COVID-19.

## Introduction

The novel coronavirus disease-2019 (COVID-19), which was first reported in Wuhan, China, was declared as a public health emergency by the World Health Organization (WHO) in March 2020. The State of Qatar confirmed its first positive case on February 29, 2020. By July, Qatar had one of the highest numbers of patients with COVID-19 per million population.^1^ The pandemic puts strain on health systems and essential public services worldwide, including Qatar. Public health facilities were overwhelmed with numerous cases of COVID-19. The global response to this public health emergency varies because of different healthcare systems, policies, and resources available to countries.^2–5^ Qatar is a peninsula located amid the western coast of the Arabian Gulf. It is one of the world's wealthiest nations in terms of per capita gross domestic product and has a population of 2.7 million.^6^ Qatar launched a public health strategy and commissioned special hospitals and national quarantine sites for managing patients with COVID-19.^7^. Hospitals suspended non-urgent care and elective procedures, and healthcare workers were redeployed to COVID-19 facilities owing to the high demand at the time. This included physicians regardless of their specialty to meet the medical needs of patients with COVID-19. However, the literature on trainee redeployment during the current pandemic is scarce, mainly consisting of correspondence articles, with very little literature about psychiatrist trainee redeployment.^8,9^ Psychiatrists at all levels of clinical training encounter patients with various physical health problems, ranging from chronic medical conditions to acute medical and surgical emergencies during their daily clinical practice.^10,11^ The Accreditation Council for Graduate Medical Education (ACGME) identifies the ability of psychiatrist trainees to perform a complete basic physical examination as a mandated core clinical competency. It also mandates psychiatrist trainees to be skillful in administering all diagnostic and therapeutic procedures considered essential in their clinical practice setting.^12^ However, compared with other medical specialties, a proportion of mental healthcare professionals exhibits lower levels of self-perceived proficiency in managing physical health emergencies.^13^ Thus, this study aimed to gain insight into the attitude of psychiatrist trainees toward their foreseeable and established redeployment. This study also aimed to assess the readiness and preparedness of psychiatrist trainees to manage physical health conditions that they might encounter during redeployment. The results can help in developing appropriate training interventions for any possible redeployment of psychiatrist trainees in the future, should the need arise.

## Methods

### Setting, participants, and redeployment process

During the first wave of the pandemic in Qatar, multiple healthcare facilities were appointed as dedicated COVID-19 sites, including newly launched hospitals and upgraded general hospital wards.^7^ These were initiatives to boost the healthcare sector's inpatient and intensive care capacity to accommodate moderate and severe COVID-19 cases.

On March 31, 2020, the country's COVID-19 command center – System-Wide Incident Command Committee – notified the Department of Psychiatry, Hamad Medical Corporation (HMC), to nominate 50% of its trainees for potential redeployment to these new facilities to support the increased healthcare demands. The Department of Psychiatry in HMC has the only psychiatry residency program in Qatar, which is one of a handful of ACGE-International (ACGME-I)-accredited graduate psychiatry training programs worldwide,^14^ and hosts the majority of fellowship programs in psychiatry in the country. Trainees who were pregnant, breastfeeding, or immune, or medically compromised were exempted from redeployment. Selected eligible trainees were notified of their imminent deployment to dedicated COVID-19 sites covering moderate and severe cases and were redeployed shortly afterward. Redeployment transpired in waves from April to June 2020.

A cross-sectional survey was circulated among psychiatrist trainees (residents and fellows) at the Department of Psychiatry, HMC, Qatar. The survey took place in May 2020, during the COVID-19 outbreak in Qatar, when doctors from different departments were being deployed to work at COVID-19 sites.

### Instruments and data collection

A survey was adapted based on previous studies assessing the attitude, readiness, and preparedness of psychiatry and other noncritical care trainees and physicians-in-practice in managing acute medical emergencies.^10,11,13,15,16^

Candidate survey items were extracted from these studies and adapted to our study context. Consequently, adapted items were incorporated into our survey based on a consensus between the authors, which was achieved through unstructured group discussions (via emails and online meetings).

An email was sent to the professional email addresses of all medical residents and fellows at the Department of Psychiatry. The survey was created using Microsoft Forms (Appendix 1). To optimize the participation rates, reminders were sent on a WhatsApp group dedicated to all psychiatrist trainees in Qatar.

The survey consisted of questions about the following:

• Medical and life support training (five questions)
• Experience in managing the most common medical emergencies (seven items) and in using and interpreting the most important emergency medicine diagnostic instruments (11 questions)
• Preparation for redeployment to COVID-19 sites (eight questions)
• Perceived confidence levels in managing medical emergencies (seven questions) and in using and interpreting the most important emergency medicine diagnostic instruments (11 questions)
• Rating of trainee's preparedness to be deployed to a COVID-19 site on a 5-point Likert scale.

### Ethical Considerations

The study was considered a service evaluation project by the HMC Institutional Review Board (IRB) and was exempted from IRB approval (MPF-23-02-21). All necessary departmental approvals were obtained before commencing the study. The study was conducted in accordance with the World Medical Association Declaration of Helsinki.

### Statistical Analysis

For each categorical variable, we calculated absolute and relative frequencies. Continuous data were presented as mean and standard deviation. Mean scores of the four major questionnaire subscales – frequency of encountering physical health problems and performing emergency clinical skills, in addition to the confidence in managing and practicing them – were calculated. The internal reliability of these subscales was evaluated using Cronbach's alpha coefficient, and values >0.7 were considered acceptable.^17,18^ The normality assumption of the data was examined using the Shapiro–Wilk test. Associations between categorical variables were examined using Fisher's exact test with *post hoc* Bonferroni's adjustment for multiple comparisons. Given the nonnormality of the data, continuous variables were compared using nonparametric tests by either the Mann–Whitney U test (comparison between two groups) or the Kruskal–Wallis H test with *post hoc* Dunn–Bonferroni pairwise comparison (comparison between more than two groups), respectively. Correlations between interval and ordinal data were evaluated using Pearson's (in case of normality) and Spearman's (in case of nonnormality) correlation coefficients.

All tests were two-tailed, with a significance level α set at 0.05. All statistical analyses were performed using IBM SPSS Statistics for Windows version 26 (IBM Corp., Armonk, NY, USA). Figures were generated using OriginPro version 2021 (OriginLab Corporation, Northampton, MA, USA).

## Results

The overall response rate was 88.9% (n = 40 of 45 psychiatry trainees). As shown in Table 1, 60% (n = 24) of the respondents were residents, whereas 40% (n = 16) were fellows**.** The survey shows acceptable internal reliability in the four major subscales, with Cronbach's alpha coefficient of 0.79 for the frequency of encountering physical health problems subscale, 0.79 for the frequency of performing emergency-related clinical skills subscale, 0.85 for the confidence in managing physical health issues subscale, and 0.85 for the confidence in performing emergency-related clinical skills subscale. The total internal reliability score of these subscales was 0.91.

The mean rating for the psychiatrist trainees’ preparedness to be deployed in a COVID-19 site was 2.32, with a standard deviation of 1.14, on a scale of 1–5, with higher scores indicating better preparedness.

Most (87.5%, n = 35) of the psychiatrist trainees had less than 6 months of training in medicine over the past 5 years. Most (77.5%, n = 31) had completed an intermediate life support training in the past 5 years. Most of this training was conducted 1 and 5 years ago (Table 1).

**Figure 1.**
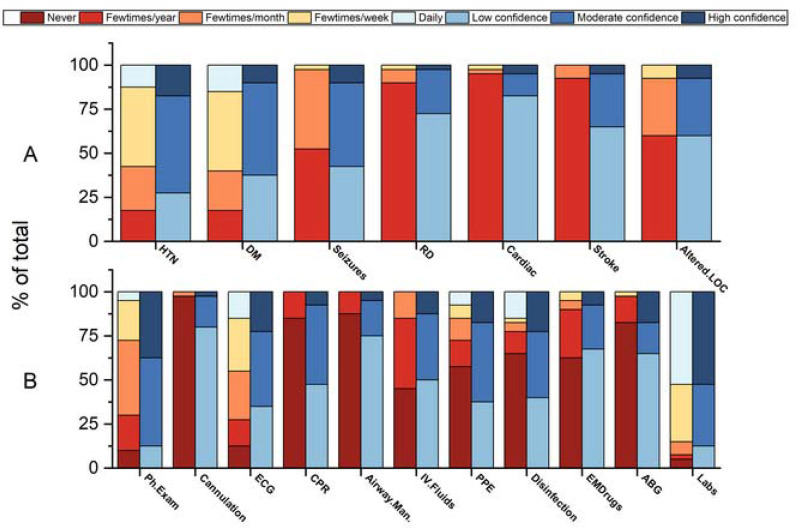
(A) Psychiatrist trainees’ frequency of encountering the most common medical emergencies and their perceived confidence levels in managing them. (B) Psychiatrist trainee's frequency of performing, using, and interpreting the most important emergency medicine diagnostic and therapeutic clinical skills and their perceived confidence levels in managing them.

Even though most psychiatrist trainees reported to commonly encounter patients with chronic medical conditions such as diabetes and hypertension, much less was reported about their experience with acute life-threatening medical conditions, such as respiratory distress, myocardial infarction, stroke, or loss of consciousness (Figure 1). Similarly, while most psychiatry trainees reported that they were commonly performing and interpreting basic laboratory tests and electrocardiography (ECG), most reported having no experience in managing airways, performing external cardiac compression, collecting arterial blood gases (ABG), or administering emergency drugs (Figure 1).

Around two-thirds of psychiatrist trainees (65%, n = 26) felt that their physical examination skills deteriorated since they started working in psychiatry (Table 1). Half of the participants felt worried or anxious about being redeployed, whereas one-quarter felt ambivalent. Only 15% (n = 6) felt honored, excited, or enthusiastic about their redeployment to COVID-19 sites (Table 2).

Although more than half of the psychiatry trainees rated their confidence levels in managing chronic medical conditions such as diabetes and hypertension as moderate, their confidence levels in managing acute life-threatening medical conditions (such as myocardial infarction, stroke, or respiratory distress) were mostly low (Figure 1).

While many psychiatry trainees were moderately confident in performing a physical examination, interpreting basic laboratory tests, or analyzing an ECG, most reported having low confidence in airway management, cannulation, administration of emergency drugs, or collection and interpretation of ABG (Figure 1). Most (87.5%, n = 35) respondents shared that they should receive further deployment training (Table 1).

As presented in Table 3, a significant association was identified between feelings toward redeployment and perception of the effect of psychiatric training on physical examination skills (*p = *0.017, Fisher's exact test). However, the *post hoc* analysis showed no significant differences between the comparison groups. Furthermore, a significant association was noted between perceiving a need for further training before redeployment and the time of being informed about the possibility of redeployment (*p* = 0.025, Fisher's exact test), with pairwise group analysis showing that only those who were informed within a week of their potential redeployment were significantly more likely to report feeling a need for further training than not (n = 7, 17.5% *vs*. n = 4, 10.0%, Bonferroni adjusted *p* = 0.005).

Table 4 illustrates the associations of trainees’ preparedness ratings for redeployment with training and redeployment-related factors. As illustrated, the level of clinical training was significantly associated with the preparedness ratings of trainees (H = 13.734, df = 6, *p* = 0.033). Nonetheless, *post hoc* adjusted comparisons revealed no significant differences in preparedness ratings across the different clinical groups.

Moreover, the time of the last medical/surgical training was significantly associated with the trainee's ratings of redeployment preparedness (H = 9.619, df = 3, *p* = 0.022). Adjusted pairwise comparisons showed that only those who had completed medical/surgical training 3–5 years earlier had significantly higher preparedness ratings than those who completed training >5 years ago (mean rank, 26.55 vs. 11.19, Dunn–Bonferroni adjusted *p* = 0.016).

Additionally, the duration of the last medical/surgical training was significantly associated with redeployment preparedness ratings (H = 15.577, df = 4, *p* = 0.004). However, *post hoc* adjusted comparisons revealed that only those who had 3–6 months of medical/surgical training during the past 5 years had significantly higher redeployment preparedness ratings than those who did not have any such training during the same period (mean rank, 27.70 vs. 9.08, Dunn–Bonferroni adjusted *p* = 0.011).

Furthermore, higher redeployment preparedness ratings were significantly associated with being informed in advance about their expected duties (mean rank, 28.54 vs. 17.05, U = 71.5, *p* = 0.003) and when redeployment was perceived to be well planned (mean rank, 24.81 vs. 16.98, U = 120.5, *p* = 0.026).

No significant associations were noted among the remaining training and redeployment-related variables, preparedness ratings, perceived need for further training, or feelings toward redeployment (Tables 3 and 4).

Tables 5–7 present the correlation matrices evaluating linear relationships among trainees’ clinical practices, their training, and various redeployment-related factors.

Overall, a significant direct correlation existed between the frequency of encountering physical health emergencies and the confidence in managing most of them (Spearman's correlation, *p* < 0.05) (Table 5). Similarly, a significant positive correlation was noted between the frequency of performing emergency-related clinical skills and the confidence in performing them (Table 6).

As displayed in Table 7, Spearman's correlation showed a significant inverse relationship between trainee's clinical level and the time elapsed since their last medical/surgical training with the frequency of encountering emergency physical health problems and performing emergency-related skills and with their confidence in performing emergency-related skills (*p* < 0.01). Only the duration of trainees’ last medical/surgical training was significantly positively correlated with their preparedness ratings (*r*_s_ = 0.34, *p* < 0.05) (Table 7).

## Discussion

In this survey about the attitude and preparedness of psychiatrist trainees in Qatar toward their redeployment to COVID-19 sites, most trainees believed that they were moderately prepared. Postgraduate medical training programs are increasingly focused on specialty; as a consequence, trainees exhibit lower levels of proficiency in managing physical health conditions. It was not surprising that most trainees reported being comfortable dealing with chronic medical conditions, but less so with acute life-threatening medical conditions. Psychiatrist trainees manage general health conditions commonly found in populations with mental illness and pursue a referral to appropriate medical care for medical emergencies because they encounter these conditions less frequently than routine conditions. Psychiatrist trainees are well versed with the medical management of patients in both inpatient and outpatient settings. They frequently encounter diabetes and hypertension cases in addition to performing and interpreting basic laboratory tests and ECGs. Half of the trainees felt moderately confident in managing chronic medical conditions. By contrast, trainees’ confidence in managing life-threatening medical conditions was low, and they reported little to no hands-on experience. These figures are in line with the evidence highlighting the limitations and challenges psychiatrist trainees face in managing emergent physical health problems compared with trainees from other acute and nonacute specialties.^13,19–21^ Half of the psychiatrist trainees reported feeling worried or anxious about their redeployment to COVID-19 sites, and most felt the need for more training before redeployment. These redeployment-related worries and concerns accorded with published reports of the emotional stance of trainees from other nonacute specialties toward their redeployment to COVID-19 frontlines, including trainees from dermatology,^22^ ophthalmology,^23–25^ otolaryngology,^26,27^ and radiology.^28^

Our analysis identified a significant association of the clinical level of trainees with the ratings of redeployment preparedness; however, a *post hoc* comparison failed to detect a significant association possibly because of a type 2 error given the small sample size. Likewise, a nonsignificant inverse correlation was noted between the advancing level of training and preparedness ratings. These findings can be explained by the higher exposure of early-stage psychiatrist trainees to physical health problems, especially during their first 2 years of training, compared with more senior trainees; since psychiatry residents are required by ACGME-I to complete rotations in various medical/surgical subspecialties during their first 2 years of training.^29^ This possibility is supported by other findings from this paper. First, higher preparedness ratings were significantly associated with the time and duration of the last medical/surgical training. Second, advancing level of training was significantly inversely correlated with the frequency of exposure to physical health problems, and the frequency of practicing emergency medicine-related clinical skills and the perceived confidence in performing them.

These results are consistent with existing literature reporting higher confidence in assessing and managing medical emergencies among psychiatrist trainees than among consultant psychiatrists, which was attributed to the lack of learning opportunities.^30^ Furthermore, there are reports of perceived deterioration of physical examination skills among psychiatrists at various levels of training since working in the field.^31^

Surprisingly, the level of redeployment preparedness or feelings toward redeployment were not significantly associated with the frequency of exposure to or confidence in managing physical health emergencies or the frequency of practicing or confidence in performing emergency medicine skills. Similarly, redeployment preparedness ratings or feelings toward redeployment was not significantly associated with the type or time since the last life support training or receiving any COVID-19-related training. Further research can determine whether this is due to the small sample size, quality of life support, or COVID-19-related training received.

To the best of our knowledge, no studies have examined the preparedness of psychiatrist trainees per se and their attitudes toward redeployment; nonetheless, we came across a study that examined the experience of redeployed physicians in training from different specialties. There have been reports about physicians in training in both Canada and France, in whom better training before redeployment was associated with better psychological outcomes.^32,33^ Residents in Canada reported dissatisfaction when they were given short notice before being redeployed, when they were not offered adequate orientation, and when they perceived gaps between what they were trained to do and the requirement of COVID-19 cases.^32^ Likewise, a survey sent to urology residents in training at 15 different departments in France revealed that “being well-trained” had a protective effect on resident burnout during the pandemic.^33^ Our study shares similar findings, as the importance of planning redeployment was also reflected in the association of the level of redeployment preparedness with being informed in advance about expected duties and when deployment is perceived as well planned.

Besides being trained or not, residents who chose voluntarily to be redeployed felt safer and more confident.^34,35^ The hours spent at work were also taken considered when redeploying residents. At the New York–Presbyterian/Columbia University Irving Medical Center, residents certainly did not exceed their maximum number of working hours. Despite the potential risk to expose more personnel to the virus, the work duration was prioritized to prevent burnout of residents and improve patient care.^8^ Various considerations were undertaken to avoid work-related stress and burnout among redeployed trainees in our study. Trainees’ typical working schedule, in most of the frontline sites in Qatar, was reduced to 8 h a day with 1 day off per week, averaging 48 working hours per week, which is nearly half of the maximum regular weekly working hours per the ACGME standards of psychiatry training programs.^12^ Furthermore, redeployed trainees were exempted from on-call duties and other after-hours clinical or healthcare-related activities during their redeployment period.

In Singapore, physicians in training redeployed to the disease epicenter were asked to rate the level of stress they experienced during redeployment and their level of resilience on a scale of 0–10, and the mean levels were 4.7 and 7.52, respectively. Interestingly, family medicine residents were the most stressed, reporting an average rating of 8.3/10.^36^ Although these results cannot be generalized, it could be argued that residents who are used to the fast-paced work in emergency settings were less stressed than residents who usually work in less acute settings.

A few studies have examined the challenges faced by residents who originally worked in non-urgent settings before being relocated to help at COVID-19 sites, as well as the different strategies followed to overcome these challenges. In the *Head & Neck Clinics* in Lombardy, Italy, as the need for more medical personnel increased, head and neck specialists, given their little training in infectious diseases, joined the treating teams as junior doctors after being provided with brief COVID-19 training.^37^ In New York, psychiatry residents were redeployed at New York–Presbyterian/Columbia University Irving Medical Center preferentially to intensive care units, to allow for closer supervision compared with working in the emergency department or in the wards.^8^ Another example of effective use of psychiatrist trainees during the COVID-19 pandemic was the same as that implemented by the New York–Presbyterian Columbia University Irving Medical Center where psychiatrist trainees participated in a psychiatry–palliative care liaison team. In this initiative, trainees provided compassionate end-of-life care.^38^ Some surgeons conquered the new challenges practically by taking small steps toward better management of acute emergencies. They started with collecting ABGs and placing nasogastric tubes, and they moved up the ladder to fulfill more challenging tasks. Their team effort was essential to their wellbeing individually.^34,35^ The realization that they were part of a bigger medical body helped them focus on the job at hand. In New York City, orthopedic surgeons explained that despite being out of their comfort zone, the majority was involved in COVID-19 teams. To prevent cross-contamination, they made sure to keep the department and COVID-19 teams separate. To maintain their mental wellbeing and support systems during this unprecedented time, they remained connected through online meetings.^39^ This also created a safe space for information dissemination and sharing of experiences for trainees and others.^9^ Regarding our trainees, various measures were put in place to ensure trainees’ psychological wellbeing during their redeployment period. The Outreach Staff Support Service, a division of Qatar's National Mental Health Helpline Service, was established during the first wave of the pandemic to proactively provide telecounseling services to frontline healthcare professionals, including redeployed trainees. This service is run by an interdisciplinary team of mental health professionals adopting the WHO mental health and psychosocial support recommendations amid the COVID-19 pandemic.^40^ Additionally, frontline healthcare workers, including redeployed trainees, had direct access to staff support clinics hosted in COVID-19 sites.^7,41^) Moreover, structural modifications were made to their working structure. Redeployed trainees functioned as a part of interdisciplinary teams composed of 4–5 trainees redeployed from various medical and surgical specialties and led by a senior clinician. This new structure has the potential of enhancing trainees’ integration into their new roles and reducing their alienation while redeployed from their original training sites.

### Strengths and limitations

To the best of our knowledge, this study is the first to examine the attitude and preparedness of psychiatrist trainees in Qatar toward redeployment during the COVID-19 pandemic. It involved all psychiatry residents and fellows, and the response rate was high (88.9%). However, our sample size was relatively small, and the possibility of a type 2 error cannot be ruled out. Our survey demonstrated high levels of internal consistency. Nevertheless, since this study was based on self-reported information, the findings are subject to response bias. The survey was also created for this study following a consensus among authors, but it was not formally pre-piloted or pre-validated before distribution to trainees. Finally, this study examined the attitudes of residents and fellows in Qatar, and the findings might not be generalizable to other countries with different health systems.

## Conclusion

COVID-19 took the world by storm and called for the healthcare systems to take unprecedented actions in response to its spread.^42^ Shortage of medical personnel and high demand in COVID-19-related clinical areas necessitated redeployment of trainees from different specialties. The prospect of working outside the usual scope of practice and environment is bound to raise feelings of anxiety. Experience from Qatar's response during the pandemic includes the novel provision of anonymous helpline services with an adjunct medical staff outreach program, in addition to staff support clinics in all COVID-19 hospitals. Additionally, providing redeployed trainees with flexible, on-call-free working schedules while engaging them as members of multidisciplinary teams helps prevent their exhaustion. Recommendations based on examples from our own and other specialties include the provision of enough notice and skills training before redeployment. We recommend deploying psychiatrist trainees to noncritical medical areas where they can contribute to the general medical care as well as to psychiatry–palliative care of patients. Once redeployed, local induction and working in teams with appropriate supervision can be helpful. Allocating “buddies” with trainees already part of the team can provide further peer support and ensure COVID-19 competency through online training.^43^ Additionally, creating a safe environment, such as online platforms, for residents and fellows to share their experiences might be beneficial.

## Figures and Tables

**Table 1:** Previous medical training of psychiatrist trainees

		n (%)

Level of clinical training	Resident PGY-1	7 (17.5%)

	Resident PGY-2	7 (17.5%)

	Resident PGY-3	4 (10.0%)

	Resident PGY-4	6 (15.0%)

	Clinical Fellow Year 1	6 (15.0%)

	Clinical Fellow Year 2	6 (15.0%)

	Clinical Fellow Year 3	4 (10.0%)

		

Time since last medical or surgical	Never	0 (0.0%)

training	Within the last year	14 (35.0%)

	1–3 years ago	7 (17.5%)

	3–5 years ago	11 (27.5%)

	>5 years ago	8 (20.0%)

		

Length of last medical or surgical	None at all	6 (15.0%)

training during the past 5 years	< 1 month	2 (5.0%)

	1–3 months	17 (42.5%)

	3–6 months	10 (25.0%)

	>6 months	5 (12.5%)

		

Type of life support training	None	2 (5.0%)

completed in the past 5 years	Basic	2 (5.0%)

	Intermediate	31 (77.5%)

	Advanced	5 (12.5%)

		

Time since last life support	Within the last year	7 (18.4%)

training, if had any	1–3 years ago	18 (47.4%)

	3–5 years ago	12 (31.6%)

	>5 years ago	1 (2.6%)

		

Feeling about physical examinationskills since starting work in psychiatry	DeterioratedRemained the sameImprovedNo	26 (65.0%)12 (30.0%)2 (5.0%)5 (12.5%)


**Table 2:** Preparation for deployment to COVID-19 sites

		n (%)

**Received inquiry from the residency program about**	Yes	33 (82.5%)

their health status for readiness for redeployment	No	7 (17.5%)

**Received training resources for management of**	Yes	15 (37.5%)

COVID-19-related medical problems	No	11 (27.5%)

	I do not know	14 (35.0%)

**Informed about expected duties in redeployment**	Yes	12 (30.0%)

sites	No	28 (70.0%)

**Received any COVID-19-related skills-based**	Yes	5 (12.5%)

**training in preparation for redeployment**	No	35 (87.5%)

**Awareness of the “Buddy Program” in preparation**	Yes	0 (0.0%)

**for COVID-19 redeployment**	No	40 (100.0%)

**Time first informed about the possibility**	Within a week	11 (27.5%)

**of being redeployed**	Within a month	19 (47.5%)

	More than a month	10 (25.0%)

**Feeling possible redeployment is well planned**	Yes	18 (45.0%)

**given the unprecedented circumstances**	No	22 (55.0%)

**Emotional state in anticipation of being redeployed**	Ambivalent	10 (25.0%)

	Anxious or worried	20 (50.0%)

	Enthusiastic/excitedHonoredUninterestedAngry	3 (7.5%)3 (7.5%)4 (10.0%)0 (0.0%)


**Table 3:** Results of Fisher's exact test for associations between perceived need for further training and feelings toward redeployment with clinical training and redeployment-related factors

	Feel should receive further training				Feelings toward redeployment					

		Yes	No		Ambivalent	Anxious or worried	Enthusiastic or excited	Honored	Uninterested	

		n (%)	n (%)	*p*-value	n (%)	n (%)	n (%)	n (%)	n (%)	*p*-value

Level of clinical training	Resident Year 1	7 (17.5%)	0 (0.0%)	0.618	2 (5.0%)	4 (10.0%)	0 (0.0%)	1 (2.5%)	0 (0.0%)	0.551

	Resident Year 2	5 (12.5%)	2 (5.0%)		2 (5.0%)	2 (5.0%)	1 (2.5%)	2 (5.0%)	0 (0.0%)	

	Resident Year 3	4 (10.0%)	0 (0.0%)		1 (2.5%)	3 (7.5%)	0 (0.0%)	0 (0.0%)	0 (0.0%)	

	Resident Year 4	6 (15.0%)	0 (0.0%)		2 (5.0%)	4 (10.0%)	0 (0.0%)	0 (0.0%)	0 (0.0%)	

	Fellow Year 1	5 (12.5%)	1 (2.5%)		1 (2.5%)	3 (7.5%)	1 (2.5%)	0 (0.0%)	1 (2.5%)	

	Fellow Year 2	5 (12.5%)	1 (2.5%)		0 (0.0%)	2 (5.0%)	1 (2.5%)	0 (0.0%)	3 (7.5%)	

	Fellow Year 3	3 (7.5%)	1 (2.5%)		2 (5.0%)	2 (5.0%)	0 (0.0%)	0 (0.0%)	0 (0.0%)	

**Time since the last medical or surgical training **	Within the last year	13 (32.5%)	1 (2.5%)	0.687	3 (7.5%)	7 (17.5%)	1 (2.5%)	2 (5.0%)	1 (2.5%)	0.931

	1–3 years ago	6 (15.0%)	1 (2.5%)		2 (5.0%)	4 (10.0%)	0 (0.0%)	1 (2.5%)	0 (0.0%)	

3–5 years ago	10 (25.0%)	1 (2.5%)		3 (7.5%)	5 (12.5%)	2 (5.0%)	0 (0.0%)	1 (2.5%)	

> 5 years ago	6 (15.0%)	2 (5.0%)		2 (5.0%)	4 (10.0%)	0 (0.0%)	0 (0.0%)	2 (5.0%)	

**Duration of the last medical or surgical training**	None at all	4 (10.0%)	2 (5.0%)	0.604	1 (2.5%)	2 (5.0%)	0 (0.0%)	0 (0.0%)	3 (7.5%)	0.289

	< 1 month	2 (5.0%)	0 (0.0%)		0 (0.0%)	2 (5.0%)	0 (0.0%)	0 (0.0%)	0 (0.0%)	

1–3 months	15 (37.5%)	2 (5.0%)		5 (12.5%)	7 (17.5%)	3 (7.5%)	1 (2.5%)	1 (2.5%)	

3–6 months	9 (22.5%)	1 (2.5%)		4 (10.0%)	5 (12.5%)	0 (0.0%)	1 (2.5%)	0 (0.0%)	

> 6 months	5 (12.5%)	0 (0.0%)		0 (0.0%)	4 (10.0%)	0 (0.0%)	1 (2.5%)	0 (0.0%)	

**Type of life support training in the past 5 years**	None	2 (5.0%)	0 (0.0%)	0.742	0 (0.0%)	1 (2.5%)	0 (0.0%)	0 (0.0%)	1 (2.5%)	0.559

	Basic	2 (5.0%)	0 (0.0%)		0 (0.0%)	1 (2.5%)	1 (2.5%)	0 (0.0%)	0 (0.0%)	

Intermediate	27 (67.5%)	4 (10.0%)		9 (22.5%)	15 (37.5%)	2 (5.0%)	2 (5.0%)	3 (7.5%)	

Advanced	4 (10.0%)	1 (2.5%)		1 (2.5%)	3 (7.5%)	0 (0.0%)	1 (2.5%)	0 (0.0%)	

**Time of the last life support training**	Within the last year	6 (15.8%)	1 (2.6%)	0.859	4 (10.5%)	1 (2.6%)	0 (0.0%)	1 (2.6%)	1 (2.6%)	0.124

	1–3 years ago	15 (39.5%)	3 (7.9%)		4 (10.5%)	12 (31.6%)	0 (0.0%)	1 (2.6%)	1 (2.6%)	

3–5 years ago	11 (28.9%)	1 (2.6%)		2 (5.3%)	5 (13.2%)	3 (7.9%)	1 (2.6%)	1 (2.6%)	

> 5 years ago	1 (2.6%)	0 (0.0%)		0 (0.0%)	1 (2.6%)	0 (0.0%)	0 (0.0%)	0 (0.0%)	

**Perception of physical examination skills since starting psychiatry training**	Deteriorated	22 (55.0%)	4 (10.0%)	1	9 (22.5%)	12 (30.0%)	2 (5.0%)	2 (5.0%)	1 (2.5%)	0.017

Remained the same	11 (27.5%)	1 (2.5%)		1 (2.5%)	8 (20.0%)	0 (0.0%)	0 (0.0%)	3 (7.5%)	

Improved	2 (5.0%)	0 (0.0%)		0 (0.0%)	0 (0.0%)	1 (2.5%)	1 (2.5%)	0 (0.0%)	

**Time first informed of the possibility of being redeployed**	Within a week	7 (17.5%)	4 (10.0%)	0.025	2 (5.0%)	7 (17.5%)	1 (2.5%)	0 (0.0%)	1 (2.5%)	0.821

	Within a month	18 (45.0%)	1 (2.5%)		5 (12.5%)	7 (17.5%)	2 (5.0%)	2 (5.0%)	3 (7.5%)	

	More than a month	10 (25.0%)	0 (0.0%)		3 (7.5%)	6 (15.0%)	0 (0.0%)	1 (2.5%)	0 (0.0%)	

**Informed about expected duties in redeployment sites**	Yes	9 (22.5%)	3 (7.5%)	0.149	4 (10.0%)	5 (12.5%)	2 (5.0%)	1 (2.5%)	0 (0.0%)	0.349

No	26 (65.0%)	2 (5.0%)		6 (15.0%)	15 (37.5%)	1 (2.5%)	2 (5.0%)	4 (10.0%)	

**Received COVID-19-related training resources**	I do not know	11 (27.5%)	3 (7.5%)	0.194	2 (5.0%)	9 (22.5%)	0 (0.0%)	1 (2.5%)	2 (5.0%)	0.83

Yes	15 (37.5%)	0 (0.0%)		4 (10.0%)	7 (17.5%)	2 (5.0%)	1 (2.5%)	1 (2.5%)	

No	9 (22.5%)	2 (5.0%)		4 (10.0%)	4 (10.0%)	1 (2.5%)	1 (2.5%)	1 (2.5%)	

**Received COVID-19-related skill-based training**	Yes	4 (10.0%)	1 (2.5%)	0.507	3 (7.5%)	1(2.5%)	1 (2.5%)	0 (0.0%)	0 (0.0%)	0.184

No	31 (77.5%)	4 (10.0%)		7 (17.5%)	19 (47.5%)	2 (5.0%)	3 (7.5%)	4 (10.0%)	

**Program inquired about their health status**	Yes	28 (70.0%)	5 (12.5%)	0.565	8 (20.0%)	16 (40.0%)	3 (7.5%)	3 (7.5%)	3 (7.5%)	1

No	7 (17.5%)	0 (0.0%)		2 (5.0%)	4 (10.0%)	0 (0.0%)	0 (0.0%)	1 (2.5%)	

**Feeling that their possible redeployment was well planned**	Yes	14 (35.0%)	4 (10.0%)	0.155	7 (17.5%)	7 (17.5%)	2 (5.0%)	1 (2.5%)	1 (2.5%)	0.331

No	21 (52.5%)	1 (2.5%)		3 (7.5%)	13 (32.5%)	1 (2.5%)	2 (5.0%)	3 (7.5%)	


**Bold *p* values denote significance at *p* <  0.05**

**Table 4:** Results of nonparametric tests (Mann–Whitney U and Kruskal–Wallis tests) for association between ratings of preparedness to redeployments to COVID-19 sites with clinical training and redeployment-related factors

	Rating of preparedness to be deployed to COVID-19 sites					

		n	Mean ± SD	Mean rank	H*/U**	*p*-value

Level of clinical	Resident Year 1	7	1.71 ± 0.95	14.57	13.734*	0.033

training	Resident Year 2	7	3.43 ± 0.98	30.64		

	Resident Year 3	4	2.5 ± 1	22.5		

	Resident Year 4	6	2.83 ± 0.98	25.83		

	Fellow Year 1	6	2.33 ± 1.21	20.75		

	Fellow Year 2	6	1.5 ± 0.84	12.42		

	Fellow Year 3	4	1.75 ± 0.96	14.88		

Time since the	Within the last year	14	2.21 ± 1.05	19.57	9.619*	0.022

last medical or	1–3 years ago	7	2.71 ± 1.50	23.5		

surgical training	3–5 years ago	11	2.91 ± 0.83	26.55		

	>5 years ago	8	1.38 ± 0.74	11.19		

Duration of the last medical or surgical training	None at all	6	1.17 ± 0.41	9.08	15.577*	0.004

	Less than 1 month	2	1 ± 0	7.5		

1–3 months	17	2.59 ± 0.94	23.29		

3–6 months	10	3.1 ± 1.10	27.7		

>6 months	5	1.8 ± 1.10	15.5		

Type of life support training in the past 5 years	None	2	1 ± 0	7.5	3.351*	0.341

	Basic	2	2 ± 1.41	17.5		

Intermediate	31	2.39 ± 1.15	21.06		

Advanced	5	2.6 ± 1.14	23.4		

Time of the last life support training	Within the last year	7	2.29 ± 0.95	18.57	1.997*	0.573

1–3 years ago	18	2.39 ± 1.34	19.31		

3–5 years ago	12	2.58 ± 0.9	21.42		

	>5 years ago	1	1	6.5		

Perception of physical examination skills since starting psychiatry training	Deteriorated	26	2.38 ± 1.13	21.31	1.783*	0.41

Remained the same	12	2.08 ± 1.24	17.58		

Improved	2	3.00 ± 0	27.5		

Time first informed of the possibility of being redeployed	Within a week	11	2.18 ± 1.33	19.23	0.237*	0.888

Within a month	19	2.42 ± 1.17	21.26		

More than a month	10	2.3 ± 0.95	20.45		

Informed about expected duties in redeployment sites	Yes	12	3.17 ± 1.19	28.54	71.5**	0.003

No	28	1.96 ± 0.922	17.05		

Received COVID-19-related training resources	I do not know	14	1.93 ± 1.21	16.75	5.313*	0.07

Yes	15	2.87 ± 0.99	25.63		

No	11	2.09 ± 1.04	18.27		

Received COVID-19-related skill-based training	Yes	5	2.80 ± 1.10	25.5	62.50**	0.279

No	35	2.26 ± 1.15	19.8		

Program inquired about their health status	Yes	33	2.48 ± 1.12	22.05	64.5**	0.054

No	7	1.57 ± 0.98	13.21		

Feeling that their possible redeployment was well planned	Yes	18	2.78 ± 1.22	24.81	120.5**	0.026

No	22	1.95 ± 0.95	16.98		


* Kruskal–Wallis H statistic

** Mann–Whitney U statistic

Bold *p* values denote significance at *p* < 0.05

**Table 5:** Spearman's correlation matrix for the frequency of encountering and confidence in managing physical health problems

		1	2	3	4	5	6	7	8	9	10	11	12	13	14	15	16	17

1.	Freq.HTN	1																

2.	Freq.DM	.91**	1															

3.	Freq.Seizures	.56**	.60**	1														

4.	Freq.RD	.28	.25	.33*	1													

5.	Freq.Cardiac	.25	.23	.23	.71**	1												

6.	Freq.Stroke	.26	.24	.28	.55**	.81**	1											

7.	Freq.A/LOC	.36*	.36*	.35*	.05	.24	.12	1										

8.	Mean.Health.Freq	.90**	.91**	.77**	.41**	.37*	.39*	.55**	1									

9.	Conf.HTN	.51**	.51**	.37*	.19	.21	.19	.27	.55**	1								

10.	Conf.DM	.54**	.59**	.42**	.04	-.07	-.01	.38*	.59**	.79**	1							

11.	Conf.seizures	.25	.27	.24	-.07	-.05	.01	-.04	.18	.40*	.3	1						

12.	Conf.RD	.41**	.51**	.24	-.01	.11	.03	.16	.38*	.42**	.52**	.39*	1					

13.	Conf.Cardiac	.37*	.43**	.09	.07	.19	.11	.22	.33*	.26	.41**	.33*	.63**	1				

14.	Conf.Stroke	.13	.19	.05	-.07	.07	.17	-.04	.1	.34*	.33*	.50**	.42**	.43**	1			

15.	Conf.A/LOC	.38*	.49**	.21	-.1	.03	-.05	.42**	.41**	.37*	.48**	.22	.59**	.60**	.54**	1		

16.	Mean.Health.Conf	.54**	.58**	.36*	-0.01	0.05	0.07	0.30	.53**	.79**	.79**	.66**	.70**	.60**	.67**	.70**	1	

17.	Rate.Redep.Prep	.08	.04	-.18	-.1	-.07	.01	.05	.02	.16	.08	-.02	.02	.04	.05	.16	.13	1


** Correlation is significant at the 0.01 level (two-tailed).

* Correlation is significant at the 0.05 level (two-tailed).

Abbreviations: Enc.HTN, frequency of encountering hypertension; Freq.DM, frequency of encountering diabetes mellitus; Freq.Seizures, frequency of encountering seizures; Freq.RD, frequency of encountering respiratory distress; Freq.Cardiac, frequency of encountering myocardial infarction or cardiac arrest; Freq.Stroke, frequency of encountering stroke; Freq.A/LOC, frequency of encountering altered or loss of consciousness; Mean.Health.Freq, mean score for the frequency of encountering emergency medical problems; Conf.HTN, confidence in managing hypertension; Conf.DM, confidence in managing diabetes mellitus; Conf.seizures, confidence in managing seizures; Conf.RD, confidence in managing respiratory distress; Conf.Cardiac, confidence in managing myocardial infarction or cardiac arrest; Conf.Stroke, confidence in managing stroke; Conf.A/LOC, confidence in managing altered or loss of consciousness; Mean.Health.Conf, mean score for responses assessing confidence in managing emergency medical problems; Redep.Prep, overall rating of redeployment preparedness.

**Table 6:** Combined Pearson's and Spearman's correlation matrix for the frequency of and confidence in performing the most important emergency medicine diagnostic and therapeutic technical skills

		1	2	3	4	5	6	7	8	9	10	11	12	13	14	15	16	17	18	19	20	21	22	23	24	25

1.	Freq.Physical	1																								

2.	Freq.Cannula.	.01	1																							

3.	Freq.ECG	.63**	-.05	1																						

4.	Freq.CPR	.1	-.07	.28	1																					

5.	Freq.Airway	.23	-.06	.45**	.26	1																				

6.	Freq.IVFluids	.32*	.09	.36*	.2	.17	1																			

7.	Freq.PPE	.33*	-.13	.21	.31	.22	.06	1																		

8.	Freq.Scrub	.36*	.14	.44**	.38*	.41**	.01	.59**	1																	

9.	Freq.EMDrugs	.34*	.17	.53**	.28	.24	.32*	.23	.3	1																

10.	Freq.ABG	.26	-.07	.43**	.17	.25	.22	.36*	.29	.25	1															

11.	Freq.Labs	.44**	.15	.58**	.19	.34*	.19	.05	.23	.2	.31*	1														

12.	Mean.Skills.Freq	.78**	.11	.82**	.38*	.44**	.39*	.56**	.68**	.56**	.50**	.61**	1													

13.	Conf.Physical	.38*	-.08	.55**	.27	.2	.04	.21	.3	.34*	.32*	.31*	.53**	1												

14.	Conf.Cannula	.21	.31	-.03	-.04	.00	.19	-.09	-.11	-.02	.08	.1	.1	.19	1											

15.	Conf.ECG	.43**	.04	.61**	.35*	.34*	.33*	.1	.21	.33*	.43**	.46**	.54**	.52**	.18	1										

16.	Conf.CPR	.24	.12	.31	.15	.1	.25	.07	.04	.13	.13	.22	.21	.16	.18	.41**	1									

17.	Conf.Airway	.27	-.09	.43**	.37*	.28	.14	.15	.35*	.16	.18	.17	.36*	.41**	.18	.38*	.62**	1								

18.	Conf.IVFluids	.42**	.11	.56**	.18	.22	.68**	.02	.15	.37*	.34*	.31	.51**	.3	.24	.37*	.55**	.40*	1							

19.	Conf.PPE	.26	-.19	.37*	.24	.11	.2	.46**	.42**	.14	.22	.23	.46**	.3	-.01	.24	.24	.36*	.35*	1						

20.	Conf.Scrub	.37*	-.18	.49**	.37*	.18	.22	.29	.55**	.31*	.2	.32*	.57**	.37*	.05	.28	.29	.44**	.40*	.74**	1					

21.	Conf.EMDrugs	.33*	-.11	.50**	.12	.18	.19	.07	.26	.43**	.08	.32*	.41**	.38*	-.03	.29	.2	.36*	.39*	.17	.37*	1				

22.	Conf.ABG	.19	-.11	.43**	.1	.22	.27	.07	.23	.1	.47**	.26	.32*	.23	.17	.41**	.45**	.56**	.54**	.53**	.51**	.42**	1			

23.	Conf.Labs	.46**	.15	.47**	.03	.09	.43**	.07	.08	.36*	.42**	.52**	.52**	.47**	.2	.57**	.28	.08	.35*	.29	.3	.37*	.29	1		

24.	Mean.Skills.Conf(	.48**	-.03	.70**	.33*	.26	.37*	.22	.38*	.40*	.46**	.47**	.62**(	.64**	.24	.68**	.62**	.67**	.69**	.65**	.71**	.54**	.75**	.60**	1	

25.	Rate.Redep.Prep	.06	.1	-.01	.14	-.11	.19	.08	.00	.24	-.06	-.07	.1	.3	.13	.17	.14	.19	.25	.19	.13	.32*	.02	.26	.3	1


♦ Pearson correlation

** Correlation is significant at the 0.01 level (two-tailed).

* Correlation is significant at the 0.05 level (two-tailed).

Abbreviations: Freq.Physical, frequency of performing physical examination; Freq.Cannula, frequency of performing cannulation; Freq.ECG, frequency of interpreting ECG; Freq.CPR, frequency of performing cardiopulmonary resuscitation; Freq.Airway, frequency of performing airway management; Freq.IVFluids, frequency of administering intravenous fluids; Freq.PPE, frequency of using personal protective equipment; Freq.Scrub, frequency of scrubbing and disinfection; Freq.EMDrugs, frequency of administering emergency drugs; Freq.ABG, frequency of taking arterial blood gases; Freq.Labs, frequency of interpreting basic labs; Mean.Skills.Freq, mean score of the frequency of performing emergency medicine-related clinical skills; Conf.Physical, confidence in performing physical examination; Conf.Cannula, confidence in performing cannulation; Conf.ECG, confidence in interpreting ECG; Conf.CPR, confidence in performing cardiopulmonary resuscitation; Conf.Airway, confidence in performing airway management; Conf.IVFluids, confidence in administering intravenous fluids; Conf.PPE, confidence in using personal protective equipment; Conf.Scrub, confidence in performing scrubbing and disinfection; Conf.EMDrugs, confidence in administering emergency drugs; Conf.ABG, confidence in taking arterial blood gases; Conf.Labs, confidence in interpreting basic labs; Mean.Skills.Conf, mean score of the confidence in performing emergency medicine-related clinical skills; Rate.Redep.Prep, rating of preparedness for redeployment to a COVID-19 site.

Note: All correlation results shown in the table were obtained using Spearman's correlation except those labeled with diamond superscript ♦

**Table 7:** Combined Pearson's and Spearman's correlation matrix for the training-related and redeployment-related variables

		1	2	3	4	5	6	7	8	9	10	11	12

1.	Lvl.Train	1											

2.	When.Last.Train	.79**	1										

3.	Duration.Last.Train	-.52**	-.44**	1									

4.	LS.Type	-.26	-.29	.39*	1								

5.	LS.When	.27	.09	.34*	-.04	1							

6.	Feel.PhEx	-.14	-.23	.15	.04	.16	1						

7.	When.Redep	-.37*	-.35*	.38*	.29	.11	.12	1					

8.	Mean.Health.Freq	-.46**	-.43**	.23	.24	-.16	.18	.18	1				

9.	Mean.Skills.Freq	-.53**	-.48**	.27	.23	-.19	.17	.41**	.57**♦	1			

10.	Mean.Health.Conf	-.18	-.09	-.08	.02	-.16	-.08	.07	.53**	.34*	1		

11.	Mean.Skills.Conf	-.51**	-.36*	.28	.32*	-.21	-.01	.25	.53**♦	.62**♦	.58**	1	

12.	Rate.Redep.Prep	-.2	-.13	.34*	.23	.04	-.07	.04	.02	.1	.13	.3	1


♦ Pearson correlation

** Correlation is significant at the 0.01 level (two-tailed).

* Correlation is significant at the 0.05 level (two-tailed).

Abbreviations: Lvl.Train, level of training; When.Last.Train, time of their last medical or surgical training; Duration.Last.Train, duration of their last medical or surgical training during the past 5 years; LS.Type, type of life support training they had completed in the past 5 years; LS.When, time of their last life support training; Feel.PhEx, feeling about their physical examinations skills since enrolling in psychiatry training; When.Redep, time when they were first informed about the possibility of being redeployed; Mean.Health.Freq, mean score for the frequency of encountering physical health problems; Mean.Skills.Freq, mean score of the frequency of performing emergency medicine-related clinical skills; Rate.Redep.Prep, rating of preparedness for redeployment to a COVID-19 site.

Note: All correlation values shown in the table were obtained using Spearman's correlation except those labeled with diamond superscript

**Table:** 

	Never	A few times a year	A few times a month	A few times a week	Daily

HTN	⬜	⬜	⬜	⬜	⬜

DM	⬜	⬜	⬜	⬜	⬜

Seizures	⬜	⬜	⬜	⬜	⬜

Respiratory distress	⬜	⬜	⬜	⬜	⬜

MI/cardiac arrest	⬜	⬜	⬜	⬜	⬜

Stroke	⬜	⬜	⬜	⬜	⬜

Altered/loss of consciousness	⬜	⬜	⬜	⬜	⬜


**Table:** 

	Never	A few times a year	A few times a month	A few times a week	Daily

Physical examination	⬜	⬜	⬜	⬜	⬜

Cannulation	⬜	⬜	⬜	⬜	⬜

ECG rhythm recognition	⬜	⬜	⬜	⬜	⬜

External cardiac compression	⬜	⬜	⬜	⬜	⬜

Airway management	⬜	⬜	⬜	⬜	⬜

Management of IV fluids	⬜	⬜	⬜	⬜	⬜

Proper use of PPE	⬜	⬜	⬜	⬜	⬜

Scrubbing/disinfection	⬜	⬜	⬜	⬜	⬜

Administering emergency drugs	⬜	⬜	⬜	⬜	⬜

ABG collection and interpretation	⬜	⬜	⬜	⬜	⬜

Interpreting basic lab results	⬜	⬜	⬜	⬜	⬜


**Table:** 

	Low	Moderate	High

HTN	⬜	⬜	⬜

DM	⬜	⬜	⬜

Seizures	⬜	⬜	⬜

Respiratory distress	⬜	⬜	⬜

MI/cardiac arrest	⬜	⬜	⬜

Stroke	⬜	⬜	⬜

altered/loss of consciousness	⬜	⬜	⬜


**Table:** 

	Low	Moderate	High

Physical examination	⬜	⬜	⬜

Cannulation	⬜	⬜	⬜

ECG rhythm recognition	⬜	⬜	⬜

External cardiac compression	⬜	⬜	⬜

Airway management	⬜	⬜	⬜

Management of IV fluids	⬜	⬜	⬜

Proper use of PPE	⬜	⬜	⬜

Scrubbing/disinfection	⬜	⬜	⬜

Administering emergency drugs	⬜	⬜	⬜

ABG collection and interpretation	⬜	⬜	⬜

Interpreting basic lab results	⬜	⬜	⬜


**Table:** 

1	2	3	4	5

⬜	⬜	⬜	⬜	⬜


## Appendix 1: Study survey

Attitude and Preparedness of Psychiatrist Trainees Toward Redeployment to COVID-19 Sites

### Consent

You are invited to participate in an anonymous trainee-led survey determining trainees' perceptions toward redeployment to COVID-19 sites. Do you consent on your results being utilized for research and quality improvement purposes?

Yes ⬜

No ⬜

### Training data and frequency of encountering physical health problems and performing emergency-related clinical skills

1. Please select your level of training

Resident PGY-1 ⬜

Resident PGY-2 ⬜

Resident PGY-3 ⬜

Resident PGY-4 ⬜

Clinical Fellow Year 1 ⬜

Clinical Fellow Year 2 ⬜

Clinical Fellow Year 3 ⬜

2. During your psychiatry training, when was your last training in general medical services (i.e., internal medicine, family medicine, or emergency medicine)?

Within the last year ⬜

1–3 years ago ⬜

3–5 years ago ⬜

>5 years ago ⬜

Never ⬜

3. During the past 5 years, how long did your training in general medical services last?

None at all ⬜

 < 1 month ⬜

1–3 months ⬜

3–6 months ⬜

>6 months ⬜

4. Please choose any life support training, if any, that you have done in the past 5 years

ILS ⬜

BLS ⬜

ACLS ⬜

Other ⬜

None ⬜

5. If you have had life support training, when did you have it?

Within the last year ⬜

1–3 years ago ⬜

3–5 years ago ⬜

>5 years ago ⬜

6. How regularly do you encounter the following medical issues in your psychiatry training?

7. How regularly do you perform the following skills?

8. Since starting work in psychiatry, have you felt that your physical examination skills

improved? ⬜

remained the same? ⬜

deteriorated? ⬜

### Redeployment questions

9. Has your psychiatry program inquired about your health status for readiness to be deployed?

Yes ⬜

No ⬜

10. Has your psychiatry program sent you any resource for management of COVID-19-related patients/medical problems?

Yes ⬜

No ⬜

I do not know ⬜

11. Has the organization informed you about your expected duties in the site you could be redeployed to?

Yes ⬜

No ⬜

12. Have you had any skills-based training in preparation for COVID-19-related deployment?

Yes ⬜

No ⬜

13. Are you aware of the “Buddy Program” in preparation for COVID redeployment?

Yes ⬜

No ⬜

14. When were you first informed about the possibility of being redeployed?

Within a week of being deployed ⬜

Within a month of being deployed ⬜

More than a month from being deployed ⬜

15. Do you feel your possible redeployment is well planned given the unprecedented circumstances?

Yes ⬜

No ⬜

16. What is/was your emotional state in anticipation of being redeployed?

Enthusiastic/excited ⬜

Ambivalent ⬜

Anxious/worried ⬜

Angry ⬜

Uninterested ⬜

Honored ⬜

### Confidence in managing medical emergencies and performing emergency-related clinical skills

17. Please rate your confidence level to manage the following medical issues

*High: with minimal or no supervision

*Moderate: with some supervision

*Low: with thorough supervision

18. Please rate your confidence level in performing the following skills

*High: with minimal or no supervision

*Moderate: with some supervision

*Low: with thorough supervision

19. Please rate your preparedness to be deployed to a COVID-19 site

1: Least prepared

2: Most prepared

20. Do you feel that you should receive further training before being deployed?

Yes ⬜

No ⬜
